# Low-cost dual-energy CBCT by spectral filtration of a dual focal spot X-ray source

**DOI:** 10.1038/s41598-024-60774-4

**Published:** 2024-04-30

**Authors:** Boyuan Li, Yuanming Hu, Shuang Xu, Bokuan Li, Christina R. Inscoe, Donald A. Tyndall, Yueh Z. Lee, Jianping Lu, Otto Zhou

**Affiliations:** 1 Department of Physics and Astronomy, University of North Carolina at Chapel Hill, Chapel Hill, NC 27599 USA; 2 Department of Applied Physical Sciences, University of North Carolina at Chapel Hill, Chapel Hill, NC 27599 USA; 3 Cary Academy, Cary, NC 27513 USA; 4 Department of Diagnostic Sciences, Adams School of Dentistry, University of North Carolina at Chapel Hill, Chapel Hill, NC 27599 USA; 5 Department of Radiology, University of North Carolina at Chapel Hill, Chapel Hill, NC 27599 USA

**Keywords:** Biomedical engineering, Imaging techniques and agents, Oral diseases, Imaging techniques

## Abstract

Dual-energy cone beam computed tomography (DE-CBCT) has been shown to provide more information and improve performance compared to a conventional single energy spectrum CBCT. Here we report a low-cost DE-CBCT by spectral filtration of a carbon nanotube x-ray source array. The x-ray photons from two focal spots were filtered respectively by a low and a high energy filter. Projection images were collected by alternatively activating the two beams while the source array and detector rotated around the object, and were processed by a one-step materials decomposition and reconstruction method. The performance of the DE-CBCT scanner was evaluated by imaging a water-equivalent plastic phantom with inserts containing known densities of calcium or iodine and an anthropomorphic head phantom with dental implants. A mean energy separation of 15.5 keV was achieved at acceptable dose rates and imaging time. Accurate materials quantification was obtained by materials decomposition. Metal artifacts were reduced in the virtual monoenergetic images synthesized at high energies. The results demonstrated the feasibility of high quality DE-CBCT imaging by spectral filtration without using either an energy sensitive detector or rapid high voltage switching.

## Introduction

Cone beam computed tomography (CBCT) is used extensively in medical and dental imaging, providing high isotropic resolution volumetric images at a relatively low radiation dose, cost, and footprint compared to multi-detector computed tomography (MDCT)^1–3^. Most of the current CBCT scanners acquire images using a polychromatic X-ray beam with a broad energy spectrum. Dual-energy CT (DECT) and CBCT (DE-CBCT) utilizing x-ray photons with two different energy spectra provide significantly more information than a single spectrum CT/CBCT^4–6^. They can determine the photoelectric and Compton contributions to the attenuation, synthesize virtual monoenergetic images (VMI), calculate the effective atomic number and effective electron density, reduce beam hardening artifacts, all without increasing the x-ray exposure to patients. DECT and spectral CT are increasingly used in medical imaging^7–9^.

DE-CBCT has been shown to improve the accuracy of the CT Hounsfield Unit (HU) values and bone mineral density (BMD), enable materials quantification and reduce metal artifacts by synthesizing VMIs^6,10–14^, compared to a conventional CBCT. The presence of strong metal artifacts, caused by beam hardening and photon starvation from strongly attenuating materials, is especially a pressing issue in maxillofacial imaging because of the prevalence of patients with dental restorations such as dental implants^2,15^.

Despite these promises, DE-CBCT has not been widely adopted for clinical imaging, partially due to the increased equipment cost, which can be prohibitive, especially for small dental clinics. Several methods have been reported to acquire DE-CBCT images, including using two source-detector pairs^16^, multiple x-ray tubes and a single detector^17^, kV switching^13,18^, and dual layer detector^10–12^. Spectral filtration of the X-ray source is a relatively low-cost approach for generating dual-energy X-rays. The method has been successfully implemented in a commercial DECT^19,20^, which “splits” the X-ray beam from a single source to two halves using two different x-ray filters placed adjacent to each other. It has also been investigated for DE-CBCT, by rapidly switching the filter materials placed in front of the X-ray beam to generate radiations with an alternating energy spectrum^13,21,22^. We recently proposed another source filtration-based method for dual energy imaging using an X-ray source with two focal spots and two spectral filters^23^. The x-ray photons with alternating energy spectrums are produced by rapidly switching the electron emission between the two field emission cathodes without changing the high voltage applied to the x-ray anode or moving the filters. The imaging dose from each beam can be independently programmed and optimized by changing the field emission current from each cathode and/or pulse width of each exposure. Here we report a benchtop DE-CBCT scanner designed for maxillofacial imaging using this approach. A one-step materials decomposition and reconstruction algorithm was modified specifically for this system geometry with two spatially offset X-ray focal spots. The performance of the scanner was evaluated using phantoms for materials decomposition and metal artifact reduction.

## Methods

### DE-CBCT scanner

**Figure 1:**
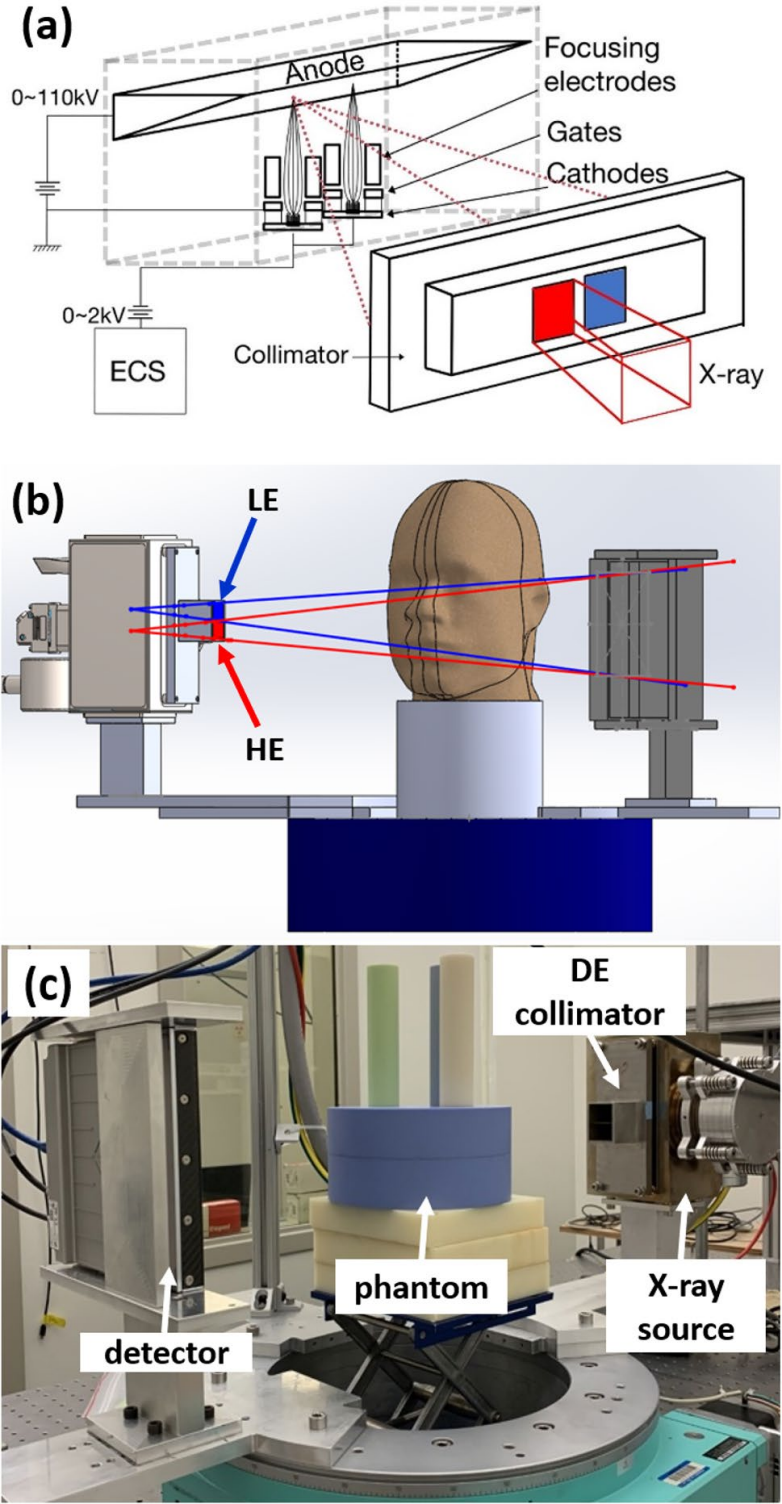
(**a**) A schematic illustration of the CNT x-ray source array. A CAD drawing (**b**) and a photo (**c**) of the benchtop DE- CBCT with a CNT x-ray source array and a flat panel detector.

The benchtop DE-CBCT scanner consists of a linear CNT x-ray source array with multiple focal spots aligned along the axial direction and a flat panel detector (FPD) mounted on a rotating gantry (Huber, Germany), as illustrated in Fig. 1. The source-object-distance (SOD) and source-imager-distance (SID) are 400 mm and 615 mm, respectively, similar to those of a commercial dental CBCT scanner. The FPD has an active area of 147.1 mm (width) × 113.7 mm (height) and a pixel pitch of 99um (Xineos-1511, Teledyne Dalsa, Waterloo, CN). To extend the FOV, the detector was shifted laterally by 70 mm to provide an effective FOV of 191 mm (width) and 70 mm (height) at the rotation axis. It was operated in the 2 × 2 binning mode. The detector integration and x-ray exposure were triggered by external triggering signals.

The CNT x-ray source array (NuRay Technologies, Chang Zhou, China) comprises multiple focal spots (“sources”) evenly distributed on an extended W anode in an evacuated stainless-steel container with a 1.7 mm thick Al window^24^. Two focal spots with a 24 mm separation were selected for this study, as illustrated in Fig. 1a. The source was operated at 110kVp. The x-ray tube current, exposure time and exposure sequence were regulated by an electronic control system (ECS, NuRay Technologies). An external collimator was designed and attached to the exit window of the x-ray source array to confine each beam to a fan angle of 13.5° degrees and cone angle of 10.1° degrees to illuminate the entire active x-ray detector area, as illustrated in Fig. 1b,c. The geometry of the scanner was calibrated by measuring a phantom with two stainless steel beads embedded in an acrylic cylinder^25^.

### Spectral filtration

A 0.15 mm Ta foil was used as the low-energy (LE) filter, and a 0.5 mm Sn as the high-energy (HE) filter, in addition to the inherent 1.7 mm Al filtration from the x-ray window. They were attached to an external collimator.

### Phantom

A 16 cm diameter water-equivalent plastic cylinder (SolidWater, Sun Nuclear Co, Melbourne FL) with four wells, and an anthropomorphic adult skull and tissue-equivalent head phantom (RANDO—radiation analogue dosimetry system; Nuclear Associates, Hicksville, NY) were imaged. A total of 7 inserts, 4 with varying concentrations of iodine (2, 5, 10 and 15 mg/mL) and 3 with calcium (50, 100, 300 mg/mL) (GAMMEX, Sun Nuclear Co, Melbourne FL) were imaged with the SolidWater phantom in two separate scans. For the RANDO phantom, a clinically used titanium implant (~ 4 mm in diameter) and a zirconia crown were added.

### Imaging protocol

For DE-CBCT imaging, 360 LE projections and 360 HE projections were collected over 360 degrees of gantry rotation by alternatively activating the LE and HE beams at the constant 110kVp x-ray tube voltage. The exposure parameters were: 110kVp, 15 mA tube current (I_tube_) and 5 ms exposure time per projection per beam (Δt_exp_). The RANDO phantom was also imaged by the same scanner operating in the regular CBCT mode using one source (referring to as “CBCT-1”) using the protocol of 110kVp, 0.3 mm Cu + 1.7 mm Al filter, 11 mA I_tube_, and 5 ms Δt_exp_, and 360 projections; and a clinical dental CBCT scanner (NT5G, New Tom, Italy, referring to as “CBCT-N”) using the standard clinical protocol for an adult patient at our institution (110 kVp, total filtration of 6 mm Al , 5 mA I_tube_, and 11.258 s total exposure time). The imaging parameters used by the 3 scanners are summarized in Table 1.

**Table 1:** Exposure parameters of the 3 scanners used in this study.

	DE-CBCT		CBCT-1	CBCT-N
	LE	HE		
Tube voltage (kVp)	110	110	110	110
Filtration	0.15 mm Ta	0.5 mm Sn	0.3 mm Cu	6 mm Al
Mean energy (keV)	60.4	75.9	62.2	56.7
Tube current(mA)	15	15	11	5
Δt_exp_ (ms)	5.0	5.0	5.0	6.5
Number of projections	360	360	360	
Total exposure (mGy)	2.37	1.42	3.94	3.78
DAP (mGy × cm^2^)	309.6	185.7	517	1090
HVL (mm Al)*	8.5	10.8	8.2	5.8
Field of view	187 mm (w) × 70 mm (h)		187 mm (w) × 70 mm (h)	180 mm (w) × 160 mm (h)

The HVL values for the DE-CBCT and CBCT-1 were experimentally measured. The value for CBCT-N was calculated using the x-ray tube voltage and the reported total filtration.

### Imaging dose

The dose profile of each beam was measured using a dose meter (Raysafe, Billdal, Sweden) placed at the center of the detector surface. The dose-area-product (DAP), a parameter commonly used to characterize the imaging dose in CBCT, was calculated for each energy spectrum by:

1 $$ \begin{array}{*{20}c} {DAP = D \times \Delta t_{\exp } \times A \times N_{view} } \\ \end{array} $$

where D is the dose rate, $\Delta t_{exp}$ is the exposure time per projection, A is the area of the detector illuminated by the primary photons, and $N_{view}$ is the number of projection views. The total DAP of the DE-CBCT scan was the sum of the values for the two energy spectra. Although not used in the imaging experiments in this paper, dynamically modulating the x-ray tube current to match the post-object dose rate between high energy and low energy was also demonstrated.

### Materials decomposition and reconstruction

The conventional projection domain material decomposition process cannot be directly applied for this system because the LE and HE projection line integrals do not coincide in space. The image domain material decomposition process is less effective in removing beam hardening and suffers from noise propagation. An advanced algorithm developed by Mechlem and coworkers^26^ that combined the reconstruction and material decomposition into one optimization problem was adopted in this study, which improves beam-hardening artifacts and reduces noise propagation. The method was originally developed for photon-counting fan-beam CT which does not require image registration. An open-source software^27^ was modified to accommodate the configuration of this DE-CBCT scanner with two offset focal spots and the cone-beam geometry.

A typical forward model based on the monochromatic Beer-Lambert’s law calculates the transmitted photon intensity $$ I_{i}$$ as:

2 $$ \begin{array}{*{20}c} {I_{i} = I_{0} exp\left( { - \mathop \sum \limits_{j = 1} A_{ij} {\upmu }_{i} } \right)} \\ \end{array} $$

where $I_{0}$ is the intensity of the incident x-ray, $A_{ij}$ is the system matrix, and $\mu_{j}$ is the linear x-ray attenuation coefficient of each image voxel. For two-material decomposition in dual-energy imaging, a polychromatic Beer-Lambert’s law is used as the forward model. The linear attenuation coefficient in each voxel $\mu_{j}$ is expressed in terms of the known mass attenuation coefficients of the two basis materials $\mu^{1,2} \left( E \right)$ and their densities $\rho_{j}^{1,2}$ and the $\rho_{j}^{1,2}$

3 $$ \begin{array}{*{20}c} {\mu_{j} \left( E \right) = \mu^{1} \left( E \right)\rho_{j}^{1} + \mu^{2} \left( E \right)\rho_{j}^{2} } \\ \end{array} $$

The transmitted photon intensity $I_{i}^{HE,LE}$ in the detector is obtained from the forward projection:

4 $$  \begin{array}{*{20}c}    {I_{i}^{{HE,LE}}  = \int\limits_{0}^{\infty } {I_{0} } S^{{HE,LE}} \left( E \right)D\left( E \right)exp\left( { - \sum\limits_{{j = 1}} {A_{{ij}} } \mu ^{{1,2}} \left( E \right)\rho _{j}^{{1,2}} } \right)}  \\   \end{array} dE  $$

where $S^{HE,LE} \left( E \right)$ is the LE/HE x-ray spectrum of the x-ray source, and $D\left( E \right)$ is the detector energy response function. The x-ray spectra $S^{HE,LE} \left( E \right)$ are simulated by the Spektr software package^28^. For the ideal energy integrated x-ray detector, one expects the detector response function to be linearly proportional to the energy. A quadratic term was added to model the non-ideal aspect of the response function as: $D\left( E \right) = E + bE^{2}$, where the empirical parameter *b* was determined by calibration using the known concentration of one of the inserts in the phantom. 

Based on the forward model, a penalized negative log-likelihood optimization problem is formed:

5 $$ \begin{array}{*{20}c} {\mathop {\text{argmin }}\limits_{{{\varvec{\rho}}^{1,2} }} \left\{ {\tilde{I} - I{\text{log}}\left( {\tilde{I}} \right) + \lambda R_{\delta } \left( x \right)} \right\}} \\ \end{array} $$

where $\tilde{I}$ is the expectation value of the detector counts, $I$ is the recorded detector counts, $R_{\delta } \left( x \right)$ is the Huber function used as regularization, $\lambda$ is the regularization strength, $\delta$ is the threshold for the Huber function and × are the material images to be computed. The Huber function is defined as:

6 $$  \begin{array}{*{20}c}    {R_{\delta } \left( x \right) = \left\{ {\begin{array}{*{20}l}    {x^{2} ,} \hfill & {if\left| x \right| \le \delta } \hfill  \\    {2\delta \left| x \right| - \delta ^{2} ,} \hfill & {otherwise} \hfill  \\   \end{array} } \right.}  \\   \end{array}  $$

A separable quadratic surrogate function derived from previous studies^29,30^ was used to estimate the cost function in the negative log-likelihood optimization problem and the second order Taylor series of the surrogate function was used for minimization. The regularization parameters were selected from a 2D parameter sweep and 300 iterations were performed. The spectrum, detector response function and reconstruction parameters were kept the same for the iodine/water basis and calcium/water basis decompositions.

Virtual monoenergetic images were synthesized from the linear combination of the materials’ mass attenuation coefficients at an energy $E$ and mass densities, $\mu_{E} = \mu_{1}^{E} \rho_{1} + \mu_{2}^{E} \rho_{2}$. The mass attenuation coefficients at a particular monoenergetic energy $\mu_{1,2}^{E}$ were obtained from the X-Ray Mass Attenuation Coefficient database maintained by the National Institute of Science and Technology (NIST).

For comparison purposes, an iterative reconstruction algorithm based on the ASTRA toolbox^31^ was used for image reconstruction of the RANDO phantom acquired with a single energy spectrum using the CBCT-1 configuration.

### Image analysis

Image quality metrics were calculated for the virtual monoenergetic images synthesized at each monoenergetic energies (keV) in section "Methods"F. The contrast-to-noise ratio (CNR) was calculated using the Hounsfield Units (HU) in the reconstructed images:

7 $$ \begin{array}{*{20}c} {CNR = \left| {HU_{Obj} - HU_{Bkg} } \right|/SD_{Bkg} } \\ \end{array} $$

where *HU*_*Obj*_ is the averaged HU value in an object region-of-interest (ROI) and *HU*_*Bkg*_ is the HU value of a homogeneous background ROI nearby. SD_Bkg_ is the standard deviation within the homogeneous background ROI nearby, which is also used to quantify noise. The metal artifact index (MAI), which has been used as a measure of the severity of the metal artifacts^13^, was calculated as:

8 $$ \begin{array}{*{20}c} {MAI = \sqrt {SD_{metal}^{2} - SD_{ref}^{2} } } \\ \end{array} $$

where *SD*_*metal*_ is the standard deviation measured from an ROI placed in the soft tissue region where metal artifacts are present and *SD*_*ref*_ is the standard deviation measured from an ROI placed in the soft tissue region without metal artifacts in the same image.

## Results

### X-ray spectra, dose rate, and imaging dose

**Figure 2:**
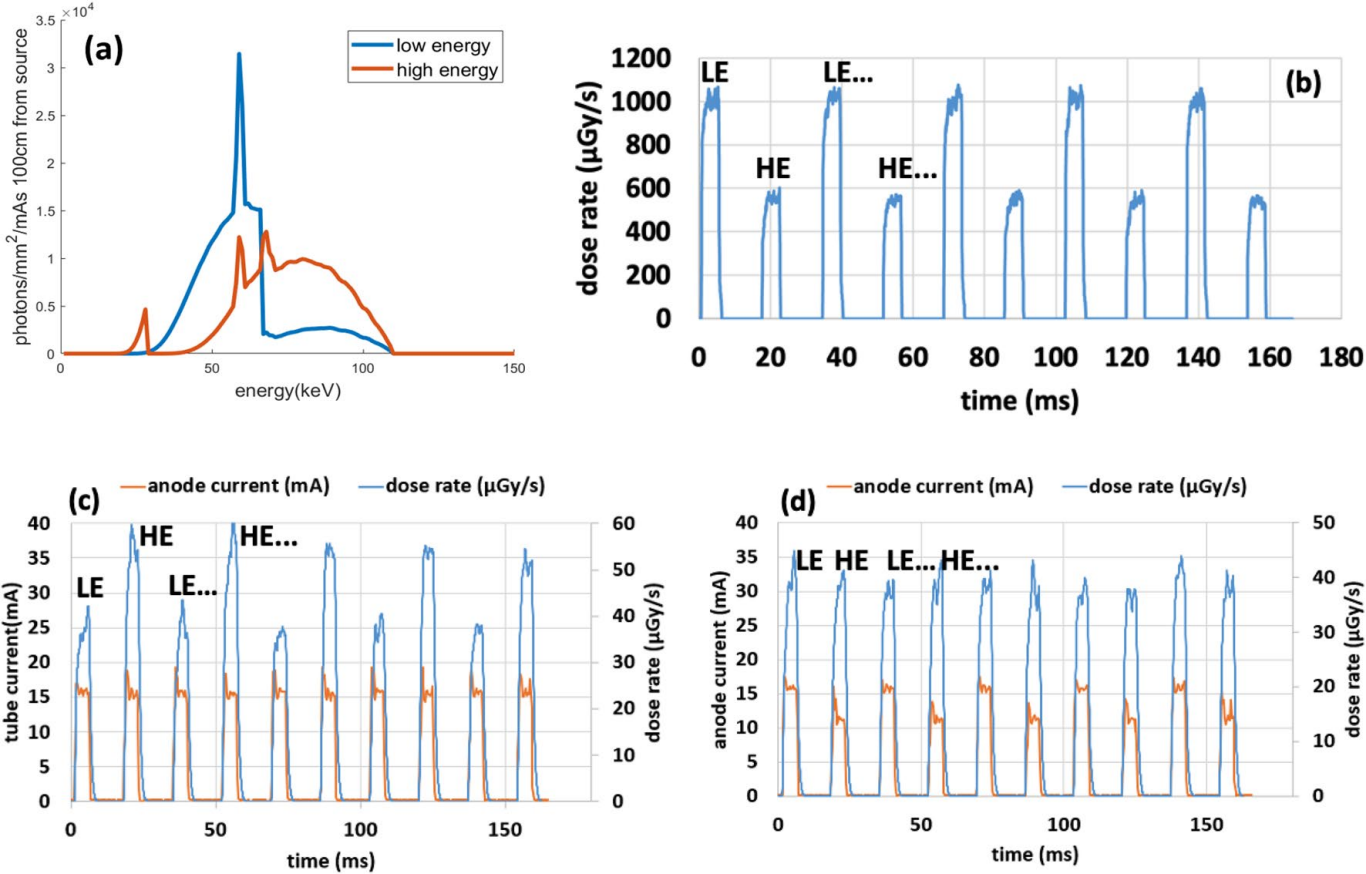
(**a**) Calculated energy spectra of the low-energy (LE) and high-energy (HE) beams at 110kVp using the open-source Spektr program^28^ (**b**) The dose profiles measured using a dosimeter placed at the detector surface without the object under the DE-CBCT imaging protocol used in this study. The rest times between the LE and HE exposures were allocated for the detector readout time. Post-object x-ray dose profiles measured using: (**c**) constant x-ray tube current for the LE and HE beams; and (**d**) modulated tube current to achieve similar post-object dose-rates.

Figure 2a shows the x-ray energy spectra of the LE and HE beams calculated using the actual experimental conditions by the open-source Spektr software package^28^. The mean energy was 60.4 keV for the LE and 75.9 keV for the HE beams. The experimentally measured dose profiles from ten consecutive exposures at alternating energies are plotted in Fig. 2b. The peak dose rate at the center of the detector surface averaged across exposures per energy was 1067µGy/s for the LE beam and 584µGy/s for the HE beam. The exposure-to-exposure variation of the imaging dose (area under each pulse) is 1.1% for the LE and 2.4% for the HE beam. The rising and falling times for each x-ray pulse are negligible. The DAP value for each energy spectrum was calculated to be 309.6 mGy*cm^2^ at LE and 185.7 mGy*cm^2^ at HE, resulting in a total DAP of 495.3 mGy*cm^2^ for a DE-CBCT scan. The corresponding imaging dose at the system isocenter was 2.37 mGy for the LE scan and 1.42 mGy for the HE scan. The corresponding half-value layers (HVL) for the LE scan and HE scan were 8.5mmAl and 10.8mmAl. The DAP was 517 mGy*cm^2^ and the peak dose rate averaged across pulses was 1670µGy/s for the regular single energy spectrum CBCT scan using CBCT-1. The corresponding imaging dose at the system isocenter was 3.94 mGy. The corresponding HVL for the CBCT-1 scan was 8.2mmAl. The results are listed in Table 1. Since the dose profiles are not perfect square profiles, an integration of the area under the dose profiles were used for computation of dose/pulse in place of the $D \times \Delta t_{exp}$ term in the DAP calculation.

The post-object dose rates after the 16 cm diameter SolidWater phantom were measured for 10 exposures with alternating low and high energies at the center of the detector and the results are shown in Fig. 2c. Because of the difference in the attenuations from the spectral filters and the phantom, the intensities of the transmitted x-rays at the two spectra are different. The dose rate difference between LE beam and HE beam is 37%. In this scanner with two independent x-ray beams, the dose allocation between the two energies can be varied by modulating the x-ray tube current or/and the exposure time for the two beams. This is demonstrated in Fig. 2d where the x-ray tube currents of the two beams were modulated at the same pulse width to achieve similar post-object dose rates where the dose rate difference is reduced to 1.3% between LE beam and HE beam. The exposure-to-exposure post-object dose variation is 5.0% for the LE and 5.3% for the HE. These are slightly larger than that for the pre-object dose, due to the significantly reduced dose rate from attenuation.

### Materials quantification

The coefficient of the quadratic term of the detector response function was determined using the concentration of one of the iodine inserts (2 mg/ml) for calibration and was found to be 0.028. The same value was then used for both the iodine-water and calcium-water decomposition.

**Figure 3:**
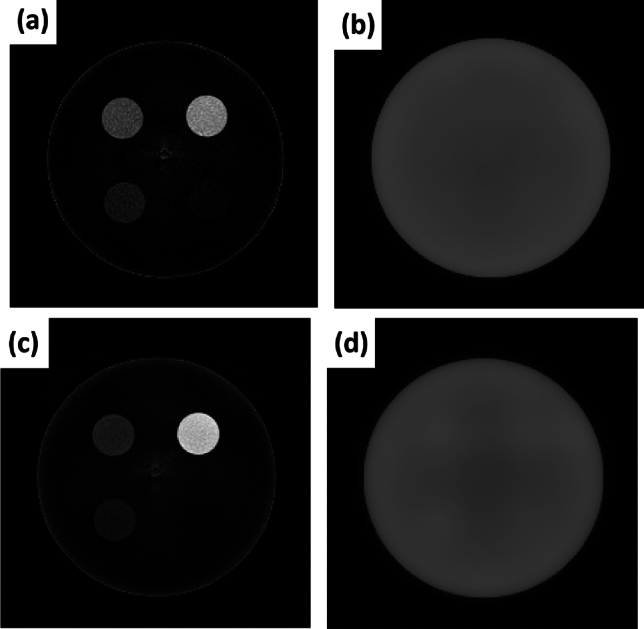
Axial slices of the iodine (**a**) and water (**b**) images of the calibration phantom from the two-materials decomposition using iodine and water as the basis; and calcium (**c**) and water (**d**) images of the phantom from the same process using calcium and water as the basis.

Figure 3 shows an axial slice of the iodine (a) and water (b) images of the SolidWater phantom with iodine inserts obtained from two base materials decomposition using iodine and water as the bases; and the calcium (c) and water (d) images of the phantom with calcium inserts using calcium and water as the two bases. The images were obtained after 300 iterations using the voxel size of 1 mm (x) × 1 mm (y) × 3 mm (z). The concentrations of the iodine inserts derived from Fig. 3a were 2.06 ± 0.78, 5.11 ± 0.75, 9.96 ± 0.83 and 14.72 ± 0.86 mg/mL for the nominal concentrations of 2, 5, 10, and 15 mg/mL; and the concentrations of the calcium inserts were 51.99 ± 7.95, 97.50 ± 7.88, and 296.38 ± 10.84 mg/mL for the nominal concentrations of 50, 100, and 300 mg/mL, respectively.

### Metal artifacts reduction and contrast

**Figure 4:**
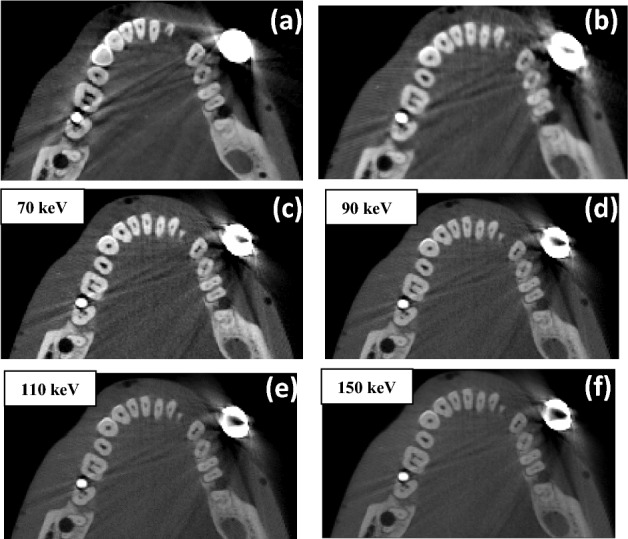
RANDO acquired with a clinical CBCT (**a**), the benchtop CBCT-1 (**b**) at 110 kV. (c,d,e,f) are VMI synthesized from the DE-CBCT dataset at 70, 90, 110 and 150 keV, respectively. Images are displayed with the same window of [-750 HU, 2250 HU].

Figure 4 shows the zoomed-in reconstructed images of the RANDO phantom from clinical CBCT-N (a) and the benchtop CBCT-1 (b) at 110 kV, and the VMI images (c-f) generated at several virtual monoenergetic energies using the DE-CBCT dataset from the DE-CBCT. Strong metal artifacts in the forms of dark halos and streaks are observed around the zirconia crown and the titanium implant with the streaks extending into regions with homogeneous materials. These are attributed to the strong attenuations from these two objects. The small circular halo around the titanium implant is attributed to the air gap between the implant and the hole in the mandible of the phantom that the implant was placed in. From visual inspection, the severities of the artifacts are reduced in the VMIs at high energies.

**Figure 5:**
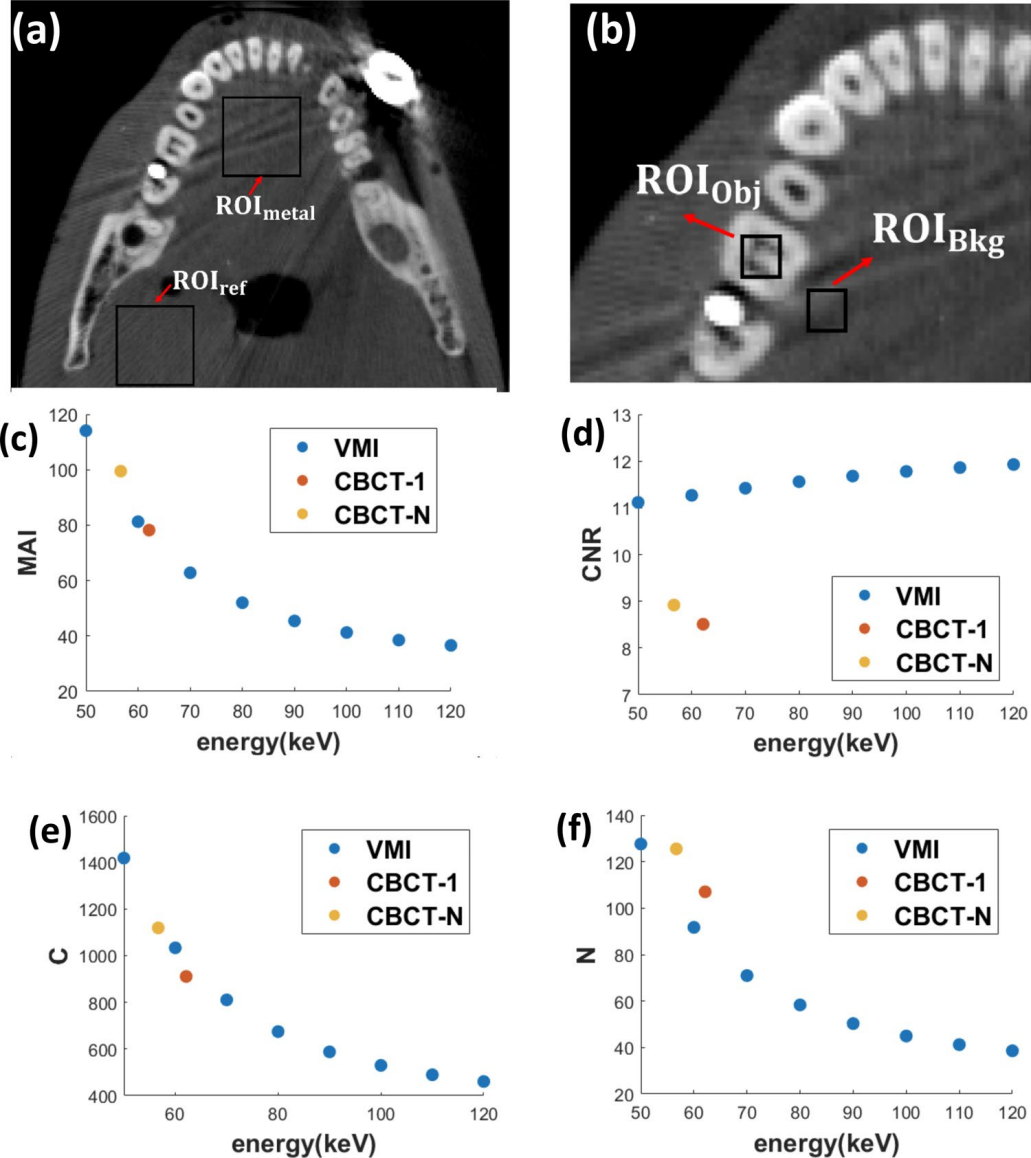
Images showing the ROIs selected for the metal artifact index (MAI) (**a**) and contrast noise ratio (CNR) (**b**) calculations. Plots of MAI (**c**), CNR (**d**), contrast (e), and noise (f) as a function of the virtual monoenergetic energy for VMIs and mean energies for CBCT-1 and CBCT-N.

The metal artifacts were quantified by calculating the MAI using the two ROIs indicated in Fig. 5a, and the CNRs were calculated using the ROIs indicated in Fig. 5b for the VMI’s in the energy range of 70–150 keV. Also included are the MAI values from the CBCT-N and CBCT-1 at their respective mean energies calculated using the respective filtration and x-ray tube energy. As shown in Fig. 5c, the MAI decreased with increasing virtual monoenergetic energy.

The MAIs of the VMIs at high energies are significantly lower than that from the CBCT-1 and CBCT-N. This is consistent with the results from previous studies using DECT^32^ and DE-CBCT^13^, and the visual observation. In Fig. 5d, the CNR’s of the VMIs are found to be higher than that of the single energy CBCT-N and CBCT-1, and increase slightly with increasing virtual monoenergetic energy. The attenuation coefficient difference between background tissue and teeth decreases with increasing energy, which explains the decrease in contrast with increasing energy in Fig. 5e. Although VMIs from DECBCT aims to remove beam-hardening artifacts, some beam-hardening artifacts remain due to system and reconstruction imperfections. As x-ray energy increases, the effects of remaining beam-hardening artifacts decrease, which explains the decrease in noise with increasing energy in Fig. 5f.

## Discussion

The results presented here demonstrate that X-ray beams with two distinct energy spectra can be generated by spectral filtration of a dual focus X-ray source for dual-energy CBCT imaging. With the set of Ta/Sn filters selected for this study, a mean energy separation of 15.5 keV between the two spectra was achieved at a fixed x-ray voltage of 110 kV, while maintaining reasonable x-ray dose rates to achieve the scanning time needed. In comparison, a mean energy separation of 15–17 keV^12,33^ at 120 kV and 9.6 keV at 80 kV^33^ were reported for dual-layer detector-based DE-CBCT. Although the energy separation can be further increased by using a stronger filtration, it will come at the expense of reduced x-ray flux and consequently increased data acquisition time. For the imaging protocol used in this study with the FPD running at 2 × 2 binning the total scanning time is 11.7 s for DE-CBCT. This is in the range of the scanning time of a single energy spectrum clinical dental CBCT^34^ and compares favorably to the value reported for a commercial dental DE-CBCT using kV switching^13^.

Phantom imaging studies were performed using the same x-ray exposure (mAs) for the two beams. Due to the differences in x-ray attenuation from the filters and the object, the post-object dose rate of the HE beam was about 70% of that of the LE beam, resulting in a higher noise level for the HE images, which may not be ideal for the overall image quality^35^. With two independently controlled cathodes and focal spots, the X-ray exposure and the dose rate of the two beams from the dual-focus x-ray source can be readily modulated in this system, as demonstrated in Fig. 2d. The effect of the dose allocation on the overall image quality will be investigated in future studies.

One drawback of using two spatially separated focal spots is the LE and HE images do not coincide. In this study a one-step materials decomposition and reconstruction algorithm was implemented for the cone beam geometry by modifying an algorithm originally developed for photon counting CT. A quadratic function was assumed for the detector response function, which was calibrated using the known mass concentration of one insert of the phantom. The same function was used in all materials decomposition calculations. The inclusion of an empirical quadratic term improved the accuracy of materials quantification compared to a linear response function. The reason is not entirely clear and can be a convolution of several factors including a small nonlinear component of the detector response and the presence of strong scatter. The latter is known to compromise the accuracy of materials quantification. No scatter subtraction algorithm was applied in this study. The accuracies of the iodine and calcium concentrations obtained are similar to those reported using a dual-layer detector-based DE-CBCT scanner^10,11^. Cupping artifacts were observed in the water images obtained from the two materials decomposition, as expected for a CBCT. They degraded the accuracies of the water densities, which were calculated to be 900 ± 30 mg/ml for the iodine phantom and 890 ± 40 mg/ml for the calcium phantom, compared to the nominal value of 1032 ± 5 mg/ml from the manufacturer.

The method described here provides DE-CBCT imaging capability using a conventional energy-integrating detector without the need for fast switching the high anode voltage. Switching between the LE and HE beams was accomplished by alternatively activating the two field emission cathodes using relatively low voltages applied between the gate and the cathode. Due to the electron field emission mechanism, the emission current and consequently x-ray generation responds instantaneously to the electron extraction voltage with minimal rising and falling times. The dual focal X-ray source is a minor modification with an insignificant added cost to a single focal spot x-ray source. As a result, the system cost is expected to be lower than the current kVp switching and dual-layer detector-based DE-CBCT design.

In principle this scanner can also be constructed using two conventional single focal spot x-ray tubes instead of a single source with two focal spots. Indeed, DE-CBCT imaging has been reported using a scanner with 3 individual X-ray tubes with one tube operating at one energy and the other two at a second energy and a common FPD^17,36^. One drawback of this approach is the reduced field of view. The relatively large inter-focal spot spacing caused by the X-ray tube vacuum housing for each tube reduces the area where x-ray beams with different energies overlap. In comparison, a relatively small inter-focal spot separation can be achieved in a CNT x-ray source array with all the spots in the same vacuum housing. In addition, the ability to manufacture source arrays with multiple focal spots has enabled a new CBCT design using multiple narrowly collimated beams to reduce scatter and cone beam artifacts^37,38^. The design can potentially be extended to dual energy imaging using multiple collimated beams for each energy to reduce scatter and improve image quality and accuracy.

## Conclusion

The results from this study demonstrated the feasibility of spectral filter-based DE-CBCT using an X-ray source with two focal spots operating at the same tube voltage. Accurate materials quantification was achieved using a one-step materials decomposition method. Metal artifact reduction was demonstrated in virtual monoenergetic images synthesized at higher energies.

## Data Availability

All data that support the findings of this study are included within the article.
